# Analysis of Landslides Triggered by October 2005, Kashmir Earthquake

**DOI:** 10.1371/currents.dis.0bc3ebc5b8adf5c7fe9fd3d702d44a99

**Published:** 2015-08-26

**Authors:** Irfan Mahmood, Shahid Nadeem Qureshi, Shahina Tariq, Luqman Atique, Muhammad Farooq Iqbal

**Affiliations:** Department of Meteorology, COMSATS Institute of Information Technology, Islamabad, Pakistan; Department of Meteorology, COMSATS Institute of Information Technology, Islamabad, Pakistan; Department of Meteorology, COMSATS Institute of Information Technology, Islamabad, Pakistan; Department of Meteorology, COMSATS Institute of Information Technology, Islamabad, Pakistan; Department of Meteorology, COMSATS Institute of Information Technology, Islamabad, Pakistan

## Abstract

Introduction: The October 2005, Kashmir earthquake main event was triggered along the Balakot-Bagh Fault which runs from Bagh to Balakot, and caused more damages in and around these areas. Major landslides were activated during and after the earthquake inflicting large damages in the area, both in terms of infrastructure and casualties. These landslides were mainly attributed to the minimum threshold of the earthquake, geology of the area, climatologic and geomorphologic conditions, mudflows, widening of the roads without stability assessment, and heavy rainfall after the earthquake. These landslides were mainly rock and debris falls. Hattian Bala rock avalanche was largest landslide associated with the earthquake which completely destroyed a village and blocked the valley creating a lake.

Discussion: The present study shows that the fault rupture and fault geometry have direct influence on the distribution of landslides and that along the rupture zone a high frequency band of landslides was triggered. There was an increase in number of landslides due to 2005 earthquake and its aftershocks and that most of earthquakes have occurred along faults, rivers and roads. It is observed that the stability of landslide mass is greatly influenced by amplitude, frequency and duration of earthquake induced ground motion. Most of the slope failures along the roads resulted from the alteration of these slopes during widening of the roads, and seepages during the rainy season immediately after the earthquake.

Conclusion: Landslides occurred mostly along weakly cemented and indurated rocks, colluvial sand and cemented soils. It is also worth noting that fissures and ground crack which were induced by main and after shock are still present and they pose a major potential threat for future landslides in case of another earthquake activity or under extreme weather conditions.

## Introduction

The Kashmir Earthquake (October 8^th^, 2005), with a moment magnitude of Mw 7.6 occurred in the northwestern part of the Himalayas. The epicenter of the earthquake (34.476^o^ N, 73.577^o^ E) was 19 km from Muzaffarabad and 105 km from Islamabad (Fig. 1). The earthquake occurred along the Balakot-Bagh Fault which is a reverse fault that runs along the right bank of Jhelum River from Muzaffarabad to Naushara and crosses the Jhelum River to the west bank. The Kashmir earthquake represents the most devastating earthquake in South Asia in recorded history.^1^ An intense aftershock sequence followed the earthquake. On 9^th^ October 2005, highest numbers of aftershocks (122) were recorded and by the end of 2005, a total of 1,778 aftershocks were recorded.^2^ Muzaffarabad and Balakot, the two most densely populated cities suffered most of the damage. The estimated death toll was around 87, 350, although it was estimated that the death toll could be over 100,000.^2^ The earthquake occurred within the Hazara-Kashmir Syntaxis along the Balakot-Bagh Fault (also known as Muzaffarabad fault) and the maximum vertical displacement on the fault was about 5 m.^3^ Thousands of very dense band of landslides were triggered as a result of the earthquake and the landslides resulted in destruction of infrastructure. The landslides were mostly triggered along the fault rupture trace. Hattian Bala rock avalanche was largest landslide associated with the earthquake which completely destroyed a village and blocked the tributaries of Jhelum River, thus creating a dam. A total of around 2500 landslides were triggered due to earthquake and mostly the landslides occurred along the hanging wall block of the fault.^4^

The greatest number of landslides occurred within sandstone and siltstone of Miocene age, Eocene and Paleocene Limestone and shale.^4^ The earthquake caused an economic loss of around 5 billion US dollars. The earthquake epicenter was located along the Kashmir Boundary Thrust (KBT) (NW-SE trending fault), which was reactivated during the Kashmir Earthquake.^5^

**Map showing major fault systems in Northern Pakistan and Kashmir Earthquake, 2005 epicenter location.**
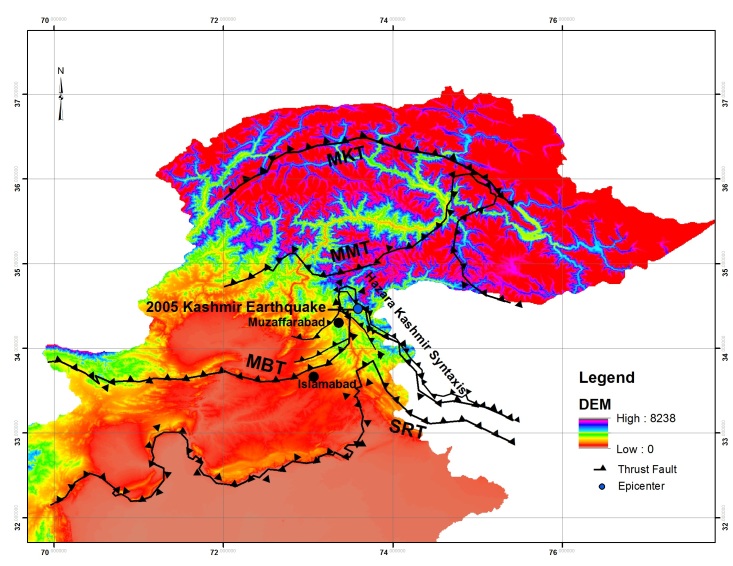
MKT= Main Karakoram Thrust, MMT= Main Mantle Thrust, MBT= Main Boundary Thrust, SRT= Salt Range Thrust

A 90 km long NW-SE belt of deformation was shown in the studies that were conducted through Synthetic Aperture Radar Data.^6^ The displacement was vertical and was from 1 m – 6 m north of Muzaffarabad.

The present case study is carried out to understand and learn the cause, vicinity areas and the impacts of landslides. Some recommendations are also made for minimizing the effects of landslides.

## Geological Setting

The study area is situated on the northwestern part of the Indian plate. The Indian plate is moving North-East at a rate of 5 cm per year and is being driven beneath Eurasian plate which is moving at a rate of 2 cm per year. Main Boundary Thrust and Panjal Thrust are folded to form an antiformal structure known as Hazara Kashmir Syntaxis (Fig. 1).^7^ The Muzaffarabad and Jhelum faults lie along the western limb of the Hazara Kashmir Syntaxis.^8^ The footwall of MBT comprises of tertiary clastics of Murree formation. Hazara Kashmir Syntaxis also comprises of siliclastics and dolomites of the Precambrian Muzaffarabad formation, Cambrian Abbottabad formation, and Paleocene Lockhart limestone and shale of patala formation.

Jhelum River and its tributaries (Neelum & Kunhar) drain the region. The rapid flow of these rivers have resulted in intense fluvial incision, thus producing steep lower valley slopes that have a gradient of > 50^o^.^9^ Between the Jhelum and Neelum river valleys, the mountains reach elevation of > 3000 m a.s.l.^10^

## Landslides

Landslides can be triggered by an earthquake, either by an increase in shear stress or due to decrease in soil strength. Significance of landslides depends upon location and magnitude of earthquakes but local conditions can also be of much importance. Earthquake triggered landslides have caused significant loss of life throughout history (Table 1).

The Kashmir earthquake triggered mass movements and these mass movements directly or indirectly caused approximately 26000 fatalities. The landslides that were triggered by the earthquake have an area of >7500 km^2^. Hazara-Kashmir syntaxis dominates the area and is enclosed by Main Boundary Thrust (MBT). The footwall of MBT is composed of Murree Formation.^11^ Other formations that are present in Hazara-Kashmir Syntaxis are Precambrian Muzaffarabad Formation (dolomites and siliclastics), Abbottabad Formation, Patala Shales and Lockhart Formation (Geological Map of the Area: Ghazanfar et al., 2010).^12^

Landslides in Murree Formation occurred alongside lower valley slopes. Landslides in Hazara Formation occurred in the fluvially incised slopes. Rockslides were extensive on the mid slope regions. Muzaffarabad Formation showed extensive fissuring.^11^

Owen et al., (2008)^11^ studied 1293 landslides and grouped them into six different geomorphic-geological settings. The study showed more than 90% of landslides were small (<1000 m^2^ in area) and were mostly in the form of shallow rock and debris falls from the top few meters of weathered bed rock and soil. Rock falls (<100 m^2^) and rock slides were common on ridge and spur crusts throughout the whole region. Slopes which have a gradient of < 20^o^ and which were present on foot wall rocks of the MBT showed little evidence of landsliding. Landslides in Murree formation occurred along lower valley slopes which have a gradient >50^o^. The study also showed that the Precambrian Dolomites and siliciclastic rocks of Muzaffarabad formation were extensively fissured. Previous studies on the effects of large earthquakes in Himalayan region were carried out by Owen et al., (1995)^13^ and Barnard et al., (2001)^14^ showed that earthquake damage was concentrated on alluvial fans and on lower stretches of valley slopes.

**Recorded large scale landslide events in terms of numbers and fatalities:** 

Location	Year	Cause of Landslide	Fatalities
Gansu, China	1920	Earthquake	180,000
Vargas, Venezuela	1999	Heavy Rainfall	30,000
TienShan, Tajikistan	1949	Earthquake	28,000
Kashmir, Pakistan	2005	Earthquake	26,500
Armero, Colombia	1985	Volcanic Eruption	23,000
Yungay, Peru	1970	Earthquake	22,000
Nevados, Huascaran	1970	Earthquake	18,000
Huaraz, Peru	1941	Landslide	4,000-6,000
Sichuan, China	1933	Earthquake	3,100
Badakshan, Afghanistan	2014	Heavy Rainfall	2,000
Rio De Janeiro, Brazil	2011	Heavy Rainfall	1,000

**Number and Percentage of different types of Landslides (Total Area = > 750 km²) (after Owen et al., 2008):** 

Type of Landslide	Number of Failures	Percentage of Total Failures
Rockfall	922	71.3
Debris fall	243	18.8
Earth fall	3	0.2
Rotational Rock Slide	14	1.1
Translational Rock Slide	39	3.0
Debris Slide	23	1.8
Rock Flow	1	0.1
Debris Flow	10	0.8
Human Induced Failures	93 Sites	53%

Landslides that were triggered by the earthquake were concentrated in specific areas that were associated with geomorphology, lithology and human factors. More than half of landslides were in some way associated with road construction and human activity (Table 2).^11^ According to Keefer (1984)^15^ and Barnard et al., (2001)^14^, the modification of landscape by humans is the most essential factor for generating landslides in tectonic areas.

The number and extent of landslides in case of Kashmir earthquake exceeds that of 1991 Garhwal earthquakes^11^ (Owen et al., 2008). This shows that a specific earthquake magnitude threshold needs to be achieved in Himalayan setting to produce landslides in wide area and this threshold was achieved during Kashmir earthquake.^11^

Monsoonal climate persists in Muzaffarabad with annual precipitation of ~1500 mm. Snow falls at altitudes of >1500 mm during winter. The fissuring that was caused by Kashmir earthquake resulted in more landsliding in 2006 during monsoon period. The debris produced as a result of landslides was re-deposited by floods resulting in landscape modification.^11^

Sato et al., (2007)^4^ used black and white 2.5 m resolution SPOT-5 satellite imagery and mapped around 2400 landslides. They noticed that more than 80% landslides were small (< 0.5 ha) and 10% of landslides were large (> 1 ha). Landslides with large area mostly occurred on steep slopes with gradient <30^o^. It was also noticed that most of the landslides occurred on the hanging wall of the fault. < 30% of the earthquakes occurred on or within 1 km from the fault. Approximately 50% of landslides occurred within 2-3 km of the fault. The highest density (3.2 landslides / km^2^) of landslides was found near Balakot in Precambrian metamorphic and sedimentary rocks whereas the second highest density (2.9 landslides / km^2^) was recorded near Muzaffarabad in Eocene and Paleocene limestone.^4^ At a distance of 10-15 km from the fault, large landslides occurred on steeper slopes (40-45^o^), whereas at much greater distance of 20 km, large landslides also occurred on gentler slopes. Half of the landslides occurred in Miocene sandstones and siltstones.^4^

The land-cover classification using ASTER satellite imagery was carried out by Kamp et al., (2008)^9^ and it showed that the landslides covered about 2.4% of the study area. The most important landslide controlling parameter was bedrock lithology. It was observed that mostly the landslides occurred in highly fractured slate, shale, dolomite, limestone and clastic sediments of the Murree and Salkhala formations (Geological Map of the Area showing landslide distribution: Kamp et al., 2008)^9^. Almost one third of the landslides occurred alongside the rivers while one fifth of the landslides occurred alongside roads. It was also observed that most of landslides after Kashmir earthquake occurred in areas which were previously marked as potentially dangerous. According to Kamp et al., (2008)^9^, almost 90% of landslides occurred at elevations below 2000 meters while only 10% occurred between 2000 and 3000 meters (Table 3). In case of most of the landslides, the slope gradient was between 25^o^– 35^o^. It was observed that the most important parameter that controls landslides is lithology. 152 Landslides occurred in Igneous Rocks formations covering a total area of 224 km^2 ^whereas 1941 landslides occurred in sedimentary rock formations covering a total area of 2292 km^2^.^9^

**The relationship of landslides (LS) to elevation within the study area of the 2005 Kashmir Earthquake (after Kamp et al., 2008):** 

Elevation (m asl)	Area (km^2^)	Area (%)	LS area (km^2^)	LS area (%)	LS area in elevation (%)
0-500	0.2	> 0.0	> 0.0	0	5.7
500-1000	311	12.2	11.5	19	3.7
1000-1500	710	27.9	29.3	48	4.1
1500-2000	667	26.2	13.0	21	1.9
2000-2500	443	17.4	3.6	6	0.8
2500-3000	263	10.3	2.4	4	0.9
3000-3500	106	4.2	1.3	2	1.2
3500-4000	35	1.4	> 0.0	0	> 0.0
4000-4446	14	0.5	> 0.0	0	> 0.0
All	2549	100	61.1	100	2.4

Areas that are underlain by Muzaffarabad, Murree and Panjal formations show highest landslide susceptibility to future failures. Formations that are in proximity to faults are also prone to future landsliding.

It is highly likely that the sites of earlier landslides may be activated and new landslides can occur along fissures especially after heavy rainfall. The ASTER 2005 Land Cover classification that was carried out by Kamp et al., (2008)^9^ showed that more than 50% of landslides (almost eight fold) occurred in grass/shrub lands which are more susceptible to landsliding. Destruction in Muzaffarabad city resulted directly from earthquake and landsliding. As a result, the alluvium of which the city is made of has a low vulnerability to future landsliding. Hazara formation has a low to moderate landslide susceptibility whereas the Murree formation has a moderate to high landslide susceptibility. Muzaffarabad formation and sites near the fault are prone to more landslides.^9^

According to Kamp et al. (2010)^16^, a six fold increase was observed in landslides from 2001 to 2009 and an eight fold increase was observed in landslide area. An inventory and susceptibility zoning map for landslides was prepared for the year 2001, which was considered as reference representing baseline landsliding. Studies conducted in 2001 and 2005 showed that the largest number of landslides occurred in Murree Formation with a nine fold increase whereas the limestone and Marble of Salkhala Formation and Panjal Formation showed second largest number of landslides with a six fold increase. Tanawal Formation showed a three fold increase in landslides with a highest increase in landslide density.

According to Sato et al., (2007)^4^ and Kamp et al., (2008)^9^; the total numbers of landslides produced as a result of Kashmir Earthquake were significantly less, as will be expected by an earthquake of similar magnitude.

Large extent of slopes were cracked within 5 km of the fault. In the years that followed, the amount of landsliding in Kashmir increased due to increase in precipitation. The rise in groundwater caused many slope failures.^1^ Many tension cracks were developed in the vicinity of the fault and have developed complex interlocking pattern of high density arrays with both bedrock and colluvium. Most of the landslides were located on the hanging wall block of the fault and landslides distribution was asymmetric. Within 5 km of the fault, large extents of slopes were cracked. Extensive slope cracking developed as a result of Kashmir earthquake. Prior to 2005 earthquake, the seismic activity in the study area was less significant due to which there were fewer cracks in slopes. After Kashmir earthquake, many tension cracks were developed in slopes. With more precipitation, the newly developed cracks will be filled with water and slope failure will occur. Extensive cracking on the hanging wall side of the fault shows that there will be many landslides in coming years.

According to Ghazanfar et al., (2010)^12^, high array of density cracks were developed on hill slopes both on hanging wall and footwall side of the fault and these cracks have the potential to fail. Through- out the Kaghan, Jhelum and Neelum valleys, the slope stability conditions were changed due to earthquake and it could result in more landsliding during monsoon periods or in case of another seismic activity.

According to Kausar et al., (2010)^10^, the earthquake resulted in deep seated and cut-slope failures and rock falls. The earthquake caused many cracks along hill slopes which are potential areas for future landslides. Landslides can occur by the increasing infiltration of water and generation of pore water pressures. Muzaffarabad, Bagh and Balakot were the largest cities that were affected by the earthquake. Landslides, rock falls, rock slides and debris flows were triggered in Jhelum, Kaghan and Neelum Valleys, some of which temporarily dammed the rivers. The white dolomites of Muzaffarabad formation failed in conjunction with red Murree formation silt and sandstones.

According to Dellow et al., (2007)^17^, the size and distribution of landslides due to Kashmir Earthquake was highly asymmetric. Three different areas of landslides can be classified;

1. The first type of landslides were formed over/adjacent to the fault rupture
2. The second type of landslides which extend about 10-20 km from fault trace were formed on hanging wall side of the fault

3. The third type of landslides were formed on the footwall side of the fault and were rare except within 2-3 km of fault trace

Studies by Saba et al. (2010)^18^ show that even before the earthquake, landslide activity was high. Although, landslides intensity increased after 2006 monsoon, the slope achieved stability afterwards and their intensity decreased.

## Hattian Bala Rock Avalanche

The Hattian area is located in Jhelum Valley. The Kashmir earthquake reactivated a landslide south of Hattian. The slide has its origin from Dana Hill in the form of huge rock avalanche. The slide blocked the tributaries of Jhelum River (Karli and Tung tributaries) creating a natural dam (View of the source of the Hattian Bala rock avalanche (Dana Hill): Dunning et al., 2007.).^19^ Dandbeh Hamlet was buried by the slide and the reported deaths were around 1000.^19^ Researchers have suggested that during the Holocene two landslides may have occurred on Dana Hill which had supposedly left two scars, one of which was the cause of the Hattian slide.

****The landslide has a total volume of approximately 85×106 m^3^; whereas, the affected area is about 1.8 km^2^. Falls and slides are a significant problem after an earthquake and they risk a population and infrastructure in lesser part of valleys.

Danna Hill lies on hanging wall of Muzaffarabad fault. The movement was favored by structurally controlled southeast plunging syncline.^20^ The deposit is mainly composed of clastic constituents of Murree formation which include sandstone, shale and mudstone. The slope failure was strongly controlled by lithology and tectonics and pre-existing rock slides. The study shows that due to inclined layering, the mudstones and sandstones of Murree formation are prone to slide.

## Discussion

The earthquake caused thousands of landslides and made many slopes unstable. Almost 26000 people lost their lives due to landslides only. The landslides were triggered by the main shock and later by aftershocks. Slope failure as a result of earthquake can only occur when a critical magnitude and peak ground acceleration is achieved as in case of Kashmir Earthquake. Muzaffarabad was the worst area affected by the earthquake. The earthquake resulted in deep seated cut slope failures and rock falls. The distribution of landslides was concentrated along precise areas related with geology and human impacts. Almost 90% of landslides were small and were in the form of rock and debris fall.^4^^,^ 9 ^,^ 11 More than half of landslides occurred in Miocene sandstones and siltstones of Murree formation.^4^^,^ 9 The dolomites and siliciclastic rocks of Muzaffarabad formation were extensively fissured. Landslides mostly occurred on steep slopes with gradient from 25^o^-35^o^.^4^^,^ 9 ^,^ 11 Major landslides occurred at elevation of around 3000 meters^9^. The Precambrian metamorphic and sedimentary rocks in the vicinity of Balakot showed highest landslide density. Muzaffarabad, Murree, Panjal formations and formations that are in proximity to the fault are prone to future landslides. Most of landslides occurred on hanging wall side of the fault and large extent of slopes were cracked in the vicinity of the fault. As 2005 was generally a dry year, groundwater was lacking and the cracked slopes could not develop into full failure but are susceptible to failure in case of another earthquake activity or under extreme weather conditions. The total number of landslides that occurred due to Kashmir Earthquake was significantly less as will be expected by a similar magnitude earthquake.^4^^,^ 9

Slope instability will increase as more forests are being converted to agricultural land and this instability during the event of an earthquake can cause landsliding. Ground shaking and structural failure were main reasons for triggering of Hattian Bala Rock avalanche. Most of landslides were small and only one the Hattian Bala landslide was major. The nature and accumulation of debris on eastern side shows that the debris moved at very high velocities.

A powerful 7.8 Mw earthquake occurred in Nepal on 25th April, 2015 and it was followed by a powerful 7.3 Mw earthquake on 12th May. Tens of thousands of landslides were triggered as a result of these two earthquakes. The landslides were triggered as far as Everest region. Many villages were affected by these landslides and hundreds of people lost their lives because of these landslides. Many more landslides can be triggered as a result of more aftershocks, gravitational failure and precipitation. Almost all of landslides were falls and slides of rock and soil. The landslides will pose serious hazard during ongoing 2015 monsoon season and 2016 monsoon season. These landslides are consistent with 2005 Kashmir earthquake landslides.^21^

## Conclusion

Kashmir earthquake caused thousands of landslides and made many slopes unstable. The Kashmir area is highly prone to landslide hazards due to its geology and structures. Mass movements can easily be triggered by slight tremors in the region. Shaking from any major future earthquake will cause liquefaction of soil, hence causing the slope to lose cohesion. Older landslides can also be activated from earthquake induced landslides. Undercutting of slopes by river erosion and human activities are the main reasons for secondary failures. More than 50% of landslides were caused by human impacts like conversion of forest land, occupancy on exposed slopes and construction of roads. The landslides patterns are quantifiably related to ground motions. Co-seismic landslides occurred at regions with steep slopes and high roughness under the influence of strong ground motion. The study show that co-seismic landslides increases in regions close to epicenter and with increasing earthquake intensity. It is also worth noting that fissures and ground crack which were induced by main shock are still present and pose a potential threat for future landslides in case of another earthquake activity or under extreme weather conditions. The debris produced as a result of landslides was re-deposited by floods resulting in major landscape modification. The landslides patterns are quantifiably related to ground motions. Co-seismic landslides occurred at regions with steep slopes and high roughness under the influence of strong ground motion. The study show that co-seismic landslides increases in regions close to epicenter and with increasing earthquake intensity. Proper identification of various types of these movements is very essential for proposing their mitigation and preventing future loss of life and property. People may be warned not to construct in the vicinity of the rupture and avoid living at the foot of the mountains. If landslides hazards are not adequately mapped and mitigated with increasing commercialization and urbanization, the problems of landslides will greatly affect life and economy.

## Competing Interests

The authors have declared that no competing interests exist.
